# Increased efficacy of dietary supplement containing wax ester-rich marine oil and xanthophylls in a mouse model of dry macular degeneration

**DOI:** 10.3389/fphar.2022.1038730

**Published:** 2022-10-13

**Authors:** Alberto Melecchi, Rosario Amato, Dominga Lapi, Massimo Dal Monte, Dario Rusciano, Paola Bagnoli, Maurizio Cammalleri

**Affiliations:** ^1^ Department of Biology, University of Pisa, Pisa, Italy; ^2^ Interdepartmental Research Center Nutrafood “Nutraceuticals and Food for Health”, University of Pisa, Pisa, Italy; ^3^ Research Center, Fidia Farmaceutici S.p.A., Catania, Italy

**Keywords:** calanus oil, omega-3 fatty acids, carotenoids, oxidative stress, inflammation, gliosis, retinal thickness

## Abstract

Age-related macular degeneration (AMD) is nowadays considered among the retinal diseases whose clinical management lacks established treatment approaches, mainly for its atrophic (dry) form. In this respect, the use of dietary patterns enriched in omega-3 and antioxidant xanthophylls has emerged as a promising approach to counteract dry AMD progression although the prophylactic potential of omega-3 of fish origin has been discussed. Whether enriched availability of omega-3 and xanthophylls may increase the effectiveness of diet supplementation in preventing dry AMD remains to be fully established. The present study aims at comparing the efficacy of an existing orally administered formulation based on lutein and fish oil, as a source of omega-3, with a novel formulation providing the combination of lutein and astaxanthin with Calanus oil (COil), which contains omega-3 together with their precursors policosanols. Using a mouse model of dry AMD based on subretinal injection of polyethylene glycol (PEG)-400, we assessed the comparative efficacy of both formulations on PEG-induced major hallmarks including oxidative stress, inflammation, glial reactivity and outer retinal thickness. Dietary supplementation with both mixtures has been found to exert a significant antioxidant and anti-inflammatory activity as reflected by the overall amelioration of the PEG-induced pathological hallmarks. Noteworthy, the formulation based on COil appeared to be more protective than the one based on fish oil, presumably because of the higher bioavailability of omega-3 in COil. These results support the use of dietary supplements combining omega-3 and xanthophylls in the prevention and treatment of AMD and suggest that the source of omega-3 might contribute to treatment efficacy.

## 1 Introduction

Age-related macular degeneration (AMD), nowadays considered as a leading cause of severe and irreversible vision impairment, includes an early phase of atrophic or dry AMD (Cabral de Guimaraes et al., 2022), which is caused by sub-retinal pigment epithelium (RPE) waste products, called drusen, that are deposited underneath the macula. Drusen accumulate over time leading to RPE dysfunction and retinal cell death, which causes early blurring with limited compromission of central vision.

Dry AMD, accounting for most of the AMD cases, is eventually followed in a minority of patients by wet (or neovascular) AMD, that is responsible of severe vision loss as weak new vessels grow behind the retina. Neovessels leak fluid and blood, thus resulting in macular edema and retinal cell death, finally leading to a rapid central vision loss (Campochiaro, 2021). In this respect, early treatment of dry AMD may potentially prevent its progression to the neovascular form, that is usually treated by intravitreal injections of anti-vascular endothelial growth factor (VEGF) molecules.

As no treatments are currently available for dry AMD, many studies are being conducted to find effective drugs to prevent the progressive growth of drusen, leading to atrophic areas within the RPE. In particular, investigations on the impact of nutrients and specific dietary patterns on the incidence and progression of AMD revealed that regular consumption of foods that contain omega-3 is associated with a lower risk of developing wet AMD (Jiang et al., 2021). In addition to omega-3 supplementation, randomized controlled trials demonstrated that treatment with antioxidants such as carotenoids may be effective against AMD progression (Cao et al., 2022). In particular, carotenoids including the xanthophylls lutein, astaxanthin and zeaxanthin accumulate in the macula and their protective efficacy is mainly related to their antioxidant, anti-inflammatory and antiapoptotic activities (Giannaccare et al., 2020). In this respect, the only option currently recommended by the American Academy of Ophthalmology is the use of antioxidant vitamins, considering their potential in slowing the progression from earlier to later stages of dry AMD (Flaxel et al., 2020).

Despite preclinical studies are indicative of the benefit of diet supplementation including fatty acids and antioxidant vitamins, evidence for their benefit to limit AMD progression remains to be fully established (de Almeida Torres et al., 2022). In this respect, diets with enriched availability of fatty acids and antioxidant xanthophylls may increase the effectiveness of diet supplementation in preventing dry AMD and therefore its progression to wet AMD.

In the present study, the polyethylene glycol (PEG)-400 model mimicking the drusen-like insult leading to dry AMD (Lyzogubov et al., 2011, 2014; Cammalleri et al., 2017; Rudolf et al., 2018) was used to assess the efficacy of an orally-administered formulation based on xanthophylls (lutein, zeaxanthin) in addition to omega-3 derived from fish oil versus a novel formulation also including astaxanthin in addition to lutein and zeaxanthin *plus* omega-3 derived from Calanus oil (COil), extracted from the marine zooplankton *Calanus finmarchicus,* mostly present in cold waters of the North. In COil, omega-3 bioavailability is increased by the presence of stearidonic acid (SDA), a precursor of eicosapentaenoic acid (EPA) and docosahexaenoic acid (DHA) Sea (Swanson et al., 2012; Cook et al., 2016). In addition, COil contains policosanols, fatty alcohols with antioxidant potential, the esterification of which may give rise to additional omega-3 including EPA and DHA (Schots et al., 2020). We tested the hypothesis that this latter enriched formulation administered before and after the PEG-400 insult, could indeed provide an increased protective efficacy in counteracting the major hallmarks of dry AMD by evaluating markers of oxidative stress, inflammation, glial activation and outer retina damage.

## 2 Materials and methods

### 2.1 Animals

Two-month-old C57BL/6J male mice were purchased from Charles River Laboratories Italia (Calco, Italy) and housed in a regulated environment (23 ± 1°C, 50 ± 5% humidity; 12 h light/dark cycles), fed with a standard diet and water *ad libitum*. A total of 48 mice were randomly assigned to 8 different groups (N = 6 each) as follows: control, PEG-injected untreated, PEG-injected treated with vehicle, PEG-injected treated with i. low or high-dose of mixture 1 (M1) or ii. low or high dose of mixture 2 (M2). For M1 and M2 composition see below. Animals used in this study were managed in agreement with the Association for Research in Vision and Ophthalmology statement for the Use of Animals in Ophthalmic and Vision Research. The present study was carried out following the European Communities Council Directive (2010/63/UE) and the Italian guidelines for animal care (DL 26/14). The experimental protocol was authorized by the Commission for Animal Wellbeing of the University of Pisa (protocol n°656/2018-PR). In accordance with the 3Rs principles for ethical use of animals in scientific research, the experimental plan was designed in order to reduce both the number and suffering of the animals.

### 2.2 Subretinal injection of polyethylene glycol

Subretinal injections of PEG-400 were performed in agreement with the protocol by Lyzogubov et al. (2014). Forty-two mice were anesthetized by intraperitoneal injection of avertin (1.2% tribromoethanol and 2.4% amylene hydrate in distilled water, 0.02 ml/g body weight: Sigma-Aldrich) before undergoing a sub-retinal injection of 0.5 mg of PEG-400 in 2 μl phosphate buffer saline (PBS) by mean of a Hamilton syringe equipped with 36-gauge needle. The formation of blebs at level of RPE/choroid complex was considered as successful injection. In order to minimize the number of the animals involved in the present study, the experimental plan did not provide a vehicle-only subretinal injection group since PBS itself does not alter cell viability or retinal structure (Lyzogubov et al., 2014).

### 2.3 Dietary supplementations

Mice were treated by oral gavage once daily 15 days before and 15 days after the sub-retinal injection of PEG-400. Twelve mice were treated with 2 different doses of M1 dissolved in sunflower oil (vehicle M1) to the final concentration of 43.8 mg/ml and administered at low dose (100 μl; 87,6 mg/kg) or high dose (200 μl; 175.2 mg/kg). M1 included fatty acids from fish oil (35.18 mg/ml of which 60% omega-3), lutein (0.88 mg/L), zeaxanthin (0.18 mg/ml), vitamin E (0.88 mg/ml), vitamin C (5.3 mg/ml), zinc (1.3 mg/ml) and copper (0.09 mg/ml). Twelve mice were treated with 2 different doses of M2 dissolved in sunflower oil *plus* tween 20 at 0.2% (vehicle M2) to the final concentration of 64 mg/ml and administered at low dose (100 μl; 128 mg/kg) or high dose (200 μl; 256 mg/kg). The M2 solution included fatty acids from COil (55.3 mg/ml of which 12% omega-3 + 33% polycosanols), astaxanthin (0.37 mg/ml), lutein (1.35 mg/ml), zeaxanthin (0.18 mg/g), vitamin E (2.7 mg/ml), vitamin C (5.3 mg/ml), zinc (1.3 mg/ml) and copper (0.09 mg/ml). In respect to fatty acids administration, despite the two mixtures display different amounts of fishery oil and Calanus oil, the actual concentration of omega 3 fatty acids delivered with the fish oil (21.5 mg/ml) is similar to that of omega 3 + polycosanols (24.5 mg/ml) delivered with Calanus oil, thus keeping the composition in fatty acids and xanthophylls as discriminant. The low doses calculated for both M1 and M2 correspond to those recommended in humans normalized by the body surface area method for interspecies’ drug dosage translation (Nair and Jacob, 2016).

### 2.4 Western blot

Fifteen days after sub-retinal PEG-400 injection, mice were sacrificed by cervical dislocation and eyes were enucleated for Western blot analysis on explanted retinas or immunofluorescence/morphometrical analysis on eye sections. Isolated retinas were lysed by sonication in RIPA lysis buffer (Santa Cruz Biotechnology, Dallas, TX, United States) containing phosphatase and proteinase inhibitors (Roche Applied Science, Indianapolis, IN, United States). Protein content was quantified by Micro BCA Protein Assay (Thermo Fisher Scientific, Waltham, MA, United States). Equal amounts of each protein sample (30 µg) were separated by SDS-PAGE (4–20%; Bio-Rad Laboratories, Inc., Hercules, CA, United States) and gels were trans-blotted onto nitrocellulose membranes (Bio-Rad Laboratories, Inc.). Membranes were blocked with 5% non-fat diet milk for 1 h at room temperature and then incubated overnight at 4°C with the solutions of primary antibodies listed in Table 1. Then, membranes were incubated for 2 h at room temperature with appropriate HRP-conjugated secondary rabbit anti-mouse (sc-2768, Santa Cruz Biotechnology) or goat anti-rabbit (170–6515, Bio-Rad Laboratories, Inc.) antibodies (1:5000). Blots were developed by the Clarity Western enhanced chemiluminescence substrate (Bio-Rad Laboratories, Inc.) and the images were acquired using the ChemiDoc XRS+ (Bio-Rad Laboratories, Inc.). The optical density (OD) relative to the target bands (Image Lab 3.0 software; Bio-Rad Laboratories, Inc.) was normalized to the corresponding OD of β-actin as loading control or nuclear factor kappa-light-chain-enhancer of activated B cells (NF-kB) p65 as appropriate.

**TABLE 1:** Western Blot primary antibodies.

Antibody	Dilution	Source	Catalogue
Rabbit monoclonal anti-GFAP	1:5000	Abcam	ab207165
Rabbit monoclonal anti-Iba-1	1:1000	Abcam	ab178846
Rabbit polyclonal anti-HO-1	1:2000	Abcam	ab13243
Rabbit monoclonal anti-NQO-1	1:10000	Abcam	ab80588
Rabbit monoclonal anti-pNF-kB p65 (Ser 536)	1:1000	Abcam	ab76302
Rabbit polyclonal anti-NF-kB p65	1:1000	Abcam	ab16502
Mouse monoclonal anti-IL-6	1:500	Santa Cruz Biotechnology	sc-57315
Rabbit monoclonal anti-TNF-α	1:1000	Abcam	ab205587
Mou se monoclonal anti-β-actin	1:2500	Sigma-Aldrich	A2228

### 2.5 Immunofluorescence and histological analysis

Enucleated eyes were fixed by immersion in 4% w/v paraformaldehyde in 0.1 M PBS for 2 h at room temperature and then stored at 4°C in 25% w/v sucrose in 0.1 M PBS. After being embedded in a cryo-gel medium, fixed eyes were cut into 10 µm thick coronal sections and mounted onto glass slides. Retinal sections corresponding to the site of lesion were selected for further immunofluorescence or histological processing.

For immunofluorescence analysis, mounted sections were incubated with the solution of primary rabbit anti-GFAP (1:400) or rabbit anti-Iba-1 (1:200) antibodies diluted in 0.1% *v/v* Triton X-100 in 0.1 M PBS overnight at 4°C. Subsequently, they were incubated with appropriate secondary goat anti-rabbit antibodies conjugated with Alexa-Fluor 488 (ab150077, Abcam; dilution: 1:200) diluted in 0.1% *v/v* Triton X-100 in 0.1 M PBS for 2 h at room temperature. Finally, retinal sections were coverslipped with Fluoroshield mounting medium containing 4′, 6-diamidino-2-phenylindole (DAPI; Abcam) and stored upon image acquisition. Images were acquired using an epifluorescence microscope (Ni-E; Nikon-Europe, Amsterdam, Netherlands) equipped with a digital camera (DS-Fi1 c; Nikon-Europe) and a ×20 plan apochromat objective. Glial fibrillary acidic protein (GFAP) and ionized calcium-binding adapter molecule (Iba)-1 immunostaining was quantified by averaging the fluorescence intensity of 5 sections corresponding to the site of the lesion in each retina (6 retinas per group). Fluorescence intensity was retrieved from grayscale images, after normalizing for the background, by measuring the mean gray level using the analysis tool of Image J (Version 1.47, NIH freeware, Bethesda, MD, United States).

For histological analysis, mounted sections were stained with hematoxylin and eosin (H&E) and images were acquired using a microscope (Ni-E; Nikon-Europe) equipped with a ×20 objective. The outer nuclear layer (ONL) thickness was considered as the interface between the outer plexiform layer and photoreceptor inner segment and quantified by averaging the values from 5 consecutive sections per retina (*n* = 6 retinas per group).

### 2.6 Statistical analysis

Statistical analysis was performed using the Graph Pad Prism 8.0.2 software (GraphPad Software, Inc., San Diego, CA, United States). Differences among groups were tested using one-way ANOVA followed by Tukey multiple comparison post hoc test. Differences with *p* < 0.05 were considered significant. All data are expressed as mean ± SEM of the indicated n values.

## 3 Results

### 3.1 Comparative efficacy of the dietary supplementations: Analysis of oxidative stress and inflammatory markers

**FIGURE 1:**
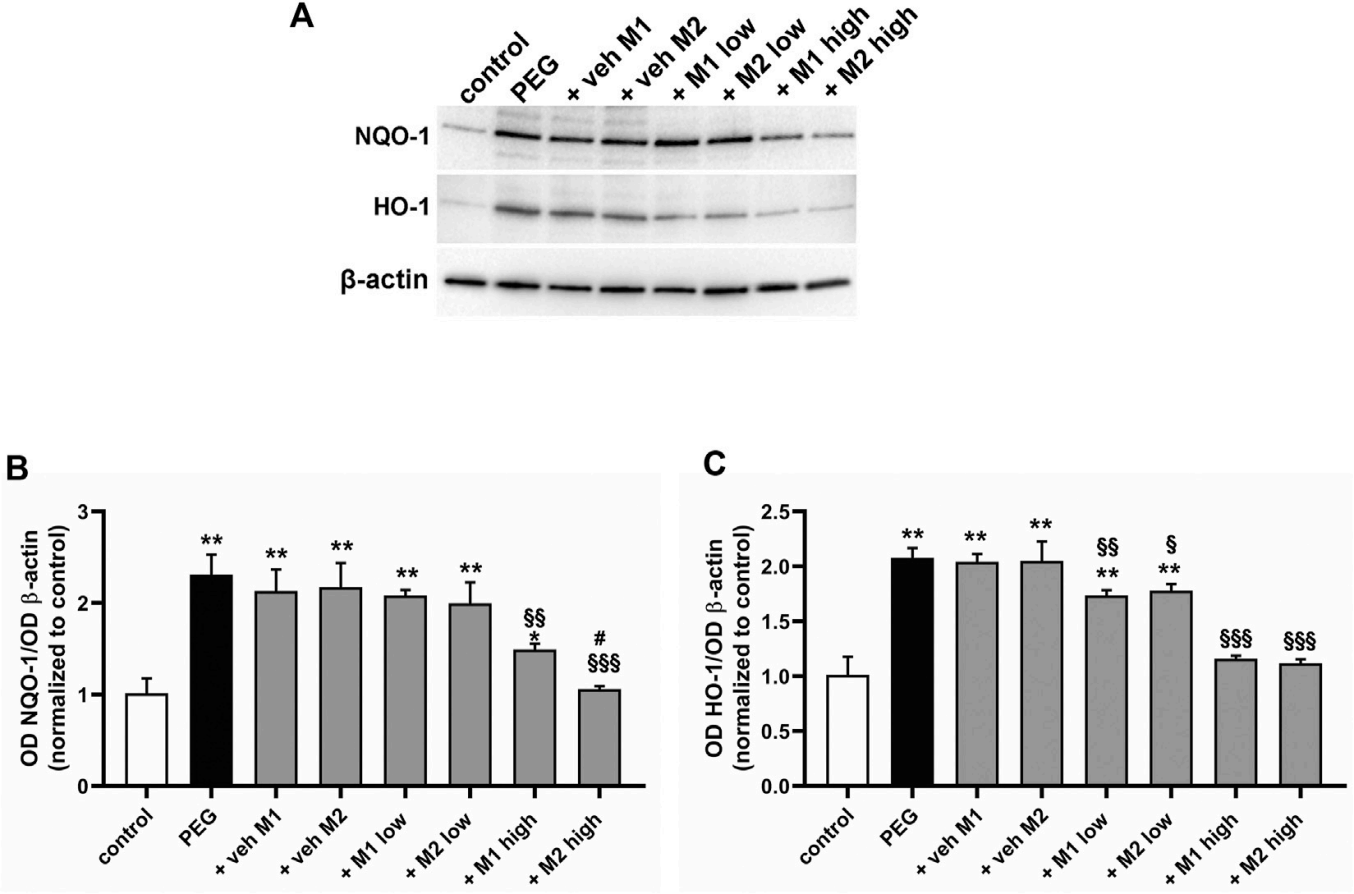
Effect of M1 and M2 on oxidative stress markers. Representative Western blots **(A)** and densitometric analysis of NQO-1 **(B)** and HO-1 **(C)** in controls, PEG-injected untreated, PEG-injected treated with M1 or M2 vehicles, PEG-injected treated with low or high dose of M1 as well as with low or high-dose of M2. β-actin was used as loading control. Data are plotted as mean ± SEM. Differences among groups were tested using one-way ANOVA followed by Tukey’s multiple comparison post-hoc test (N = 6). *p < 0.01 and **p < 0.001 vs. control; ^§^ *p* < 0.05, ^§§^p < 0.01 and ^§§§^p < 0.001 vs. respective vehicle; #*p* < 0.05 vs. M1 high.

The antioxidant efficacy of the two mixtures (M1 and M2) was evaluated by analyzing the protein levels of NAD(P)H quinone dehydrogenase-1 (NQO-1) and heme oxygenase-1 (HO-1) as major antioxidant enzymes involved in the adaptive response to altered redox balance in retinal cells (Kaspar and Jaiswal., 2009; Turpaev, 2013; Rossino et al., 2021). As shown in Figure 1, PEG-400 injection induced increased protein levels of NQO-1 and HO-1 by 115% and 103% (*p* < 0.001) respectively, as compared to control values. The oral administration of M1 and M2 vehicles did not affect the PEG-induced increase in NQO-1 and HO-1. Low doses of M1 and M2 did not prevent NQO-1 increase, whose levels were comparable to those of vehicle treated mice, while they significantly prevented the PEG-induced HO-1 increase with a 16% reduction in respect to the relative vehicles (*p* < 0.05). Treatment with high doses of both M1 and M2 significantly prevented the PEG-induced increase in NQO-1 protein levels, with M2 displaying a 23% higher efficacy as compared to the M1-treated mice. HO-1 levels in retinas of mice treated with the high dose of M1 and M2 were further decreased as compared to the respective low dose groups bringing them down to control values.

**FIGURE 2:**
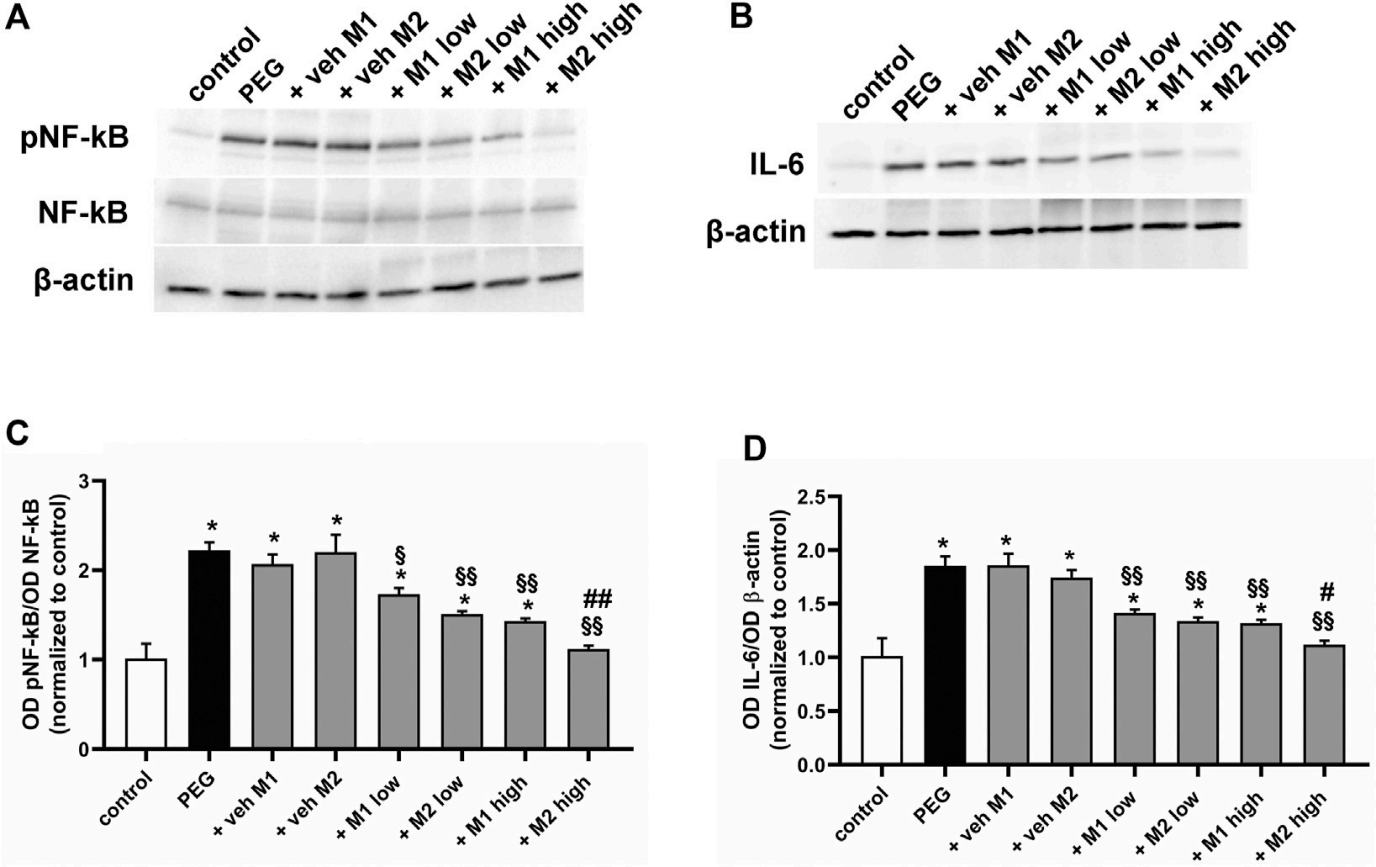
Effect of M1 and M2 on inflammatory markers. Representative Western blots **(A,B)** and densitometric analysis of pNF-kB/NF-kB ratio **(C)** and IL-6 **(D)** in controls, PEG-injected untreated, PEG-injected treated with M1 or M2 vehicles, PEG-injected treated with low or high dose of M1 as well as with low or high-dose of M2. β-actin was used as loading control. Data are plotted as mean ± SEM. Differences among groups were assessed using one-way ANOVA followed by Tukey’s multiple comparison post-hoc test (N = 6). *p < 0.001 vs. control; ^§^ *p* < 0.01 and ^§§^ *p* < 0.001 vs. respective vehicle; ^#^ *p* < 0.05, ^##^ *p* < 0.01 vs. M1 high.

The effect of the oral administration of the two compounds on PEG-induced retinal inflammation was evaluated by analyzing the phosphorylation levels of the p65 subunit of NF-kB, a key transcriptional factor regulating the proinflammatory response, and interleukin (IL)-6, a related proinflammatory cytokine (Liu et al., 2017). As shown in Figure 2, PEG injection increased NF-kB phosphorylation by 120% (*p* < 0.001) with a consequent increased production of IL-6 (+ 85%, *p* < 0.001) in respect to the control. The PEG-induced increase in pNF-kB and IL-6 was not influenced by the oral administration of either M1 or M2 vehicles. The administration of M1 and M2 at low dose significantly prevented the PEG-induced increase of both pNF-kB (−30% *p* < 0.01) and IL-6 (−25%, *p* < 0.01) in respect to vehicles, without overt differences between the two compounds. The protein levels of pNF-kB and IL-6 following the administration of M1 at high dose resulted slightly lower than those obtained in the low dose group, with a significant difference recorded exclusively for the pNF-kB levels. Conversely, the M2 high dose group displayed a more pronounced effect as compared to M1, with significantly lower levels of both pNF-kB (−22%, *p* < 0.01) and IL-6 (−17%, *p* < 0.5) compared to high dose M1 group, resulting statistically comparable to controls.

### 3.2 Comparative efficacy of the dietary supplementations: Analysis of müller cells gliosis and microglial reactivity

Among the relevant outcomes directly linked to the inflammatory activity in the retina, the glial and microglial cells reactivity is one of the main phenomena driving the retinal cell damage in AMD (Telegina et al., 2018; Rashid et al., 2019). Therefore, we analyzed the effect of the oral administration of either M1 or M2 on the transition of both Müller cells, as the glial cells mainly involved in AMD-like damage, and microglial cells to their reactive phenotypes. In particular, we analyzed the protein levels of GFAP and Iba-1 as well-established markers for reactive Müller cells and microglia, respectively, together with the immunolabeling patterns of the two markers in the PEG-induced lesion site.

**FIGURE 3:**
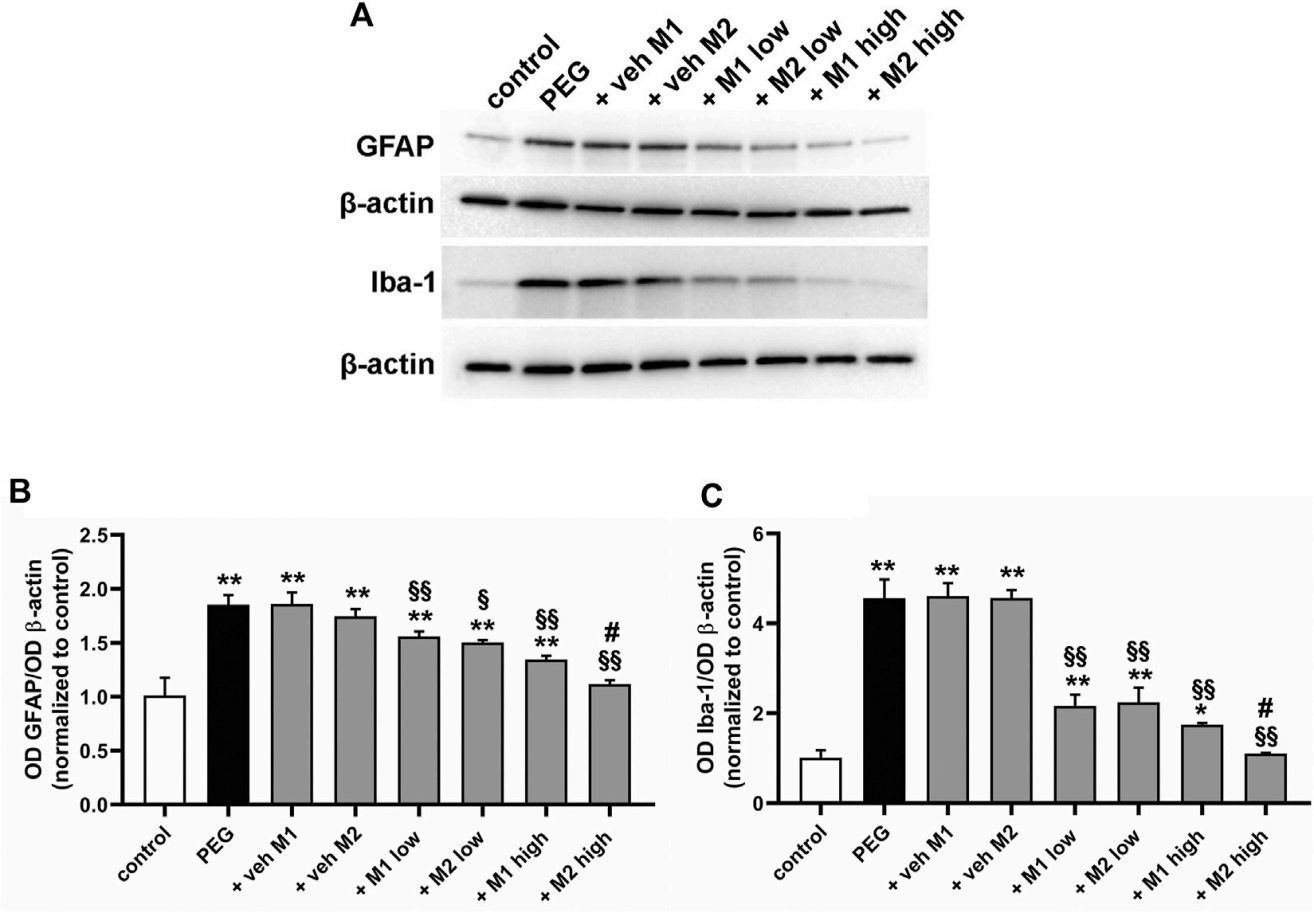
Effect of M1 and M2 on Müller cells gliosis and microglial reactivity. Representative Western blots **(A)** and densitometric analysis of GFAP **(B)** and Iba-1 **(C)** in controls, PEG-injected untreated, PEG-injected treated with M1 or M2 vehicles, PEG-injected treated with low or high dose of M1 as well as with low or high-dose of M2. β-actin was used as loading control. Data are plotted as mean ± SEM. Differences among groups were assessed using one-way ANOVA followed by Tukey’s multiple comparison post-hoc test (N = 6). *p < 0.01 and **p < 0.001 vs. control; ^§^ *p* < 0.01 and ^§§^ *p* < 0.001 vs. respective vehicle; #*p* < 0.05 vs. M1 high.

As shown in Figure 3, PEG injection significantly increased the protein levels of GFAP (+85%, *p* < 0.001) and Iba-1 (+356%, *p* < 0.001) as compared to control values. Vehicle administration did not affect GFAP and Iba-1 protein levels while low doses of both M1 and M2 comparably prevented the PEG-induced increase of GFAP (-17%, *p* < 0.05) and Iba-1 (−53%, *p* < 0.001) as compared to vehicle. Both compounds at high dose displayed a more prominent effect as compared to the low doses, with M2 resulting significantly more effective than M1 in inhibiting the PEG-induced GFAP and Iba-1 increase (−18% e −25%, respectively, *p* < 0.05).

**FIGURE 4:**
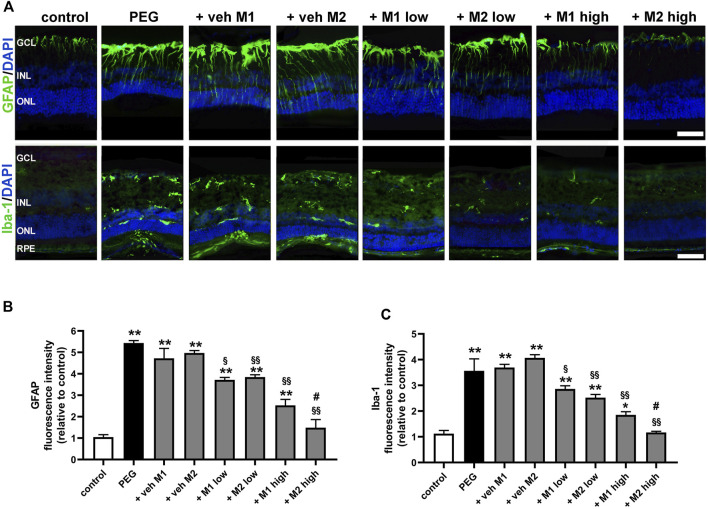
Effect of M1 and M2 on Müller cells gliosis and microglial reactivity in the site of lesion. **(A)** Representative images of retinal cross sections immunolabeled for GFAP or Iba1 in controls, PEG-injected untreated, PEG-injected treated with M1 or M2 vehicles, PEG-injected treated with low or high dose of M1 as well as with low or high-dose of M2. Scale bar, 50 μm. GCL, ganglion cell layer; INL, inner nuclear layer; ONL, outer nuclear layer; RPE, retinal pigment epithelium. Quantitative analysis of GFAP **(B)** and Iba1 **(C)** immunofluorescence intensity. Data are plotted as mean ± SEM. Differences among groups were assessed using one-way ANOVA followed by Tukey’s multiple comparison post-hoc test (N = 6). *p < 0.01 and **p < 0.001 vs. control; ^§^ *p* < 0.01, ^§§^ *p* < 0.001 vs. respective vehice; #*p* < 0.05 vs. M1 high.

As shown in Figure 4A, in control retinas, GFAP staining was limited to astrocytes localized to the ganglion cell layer (GCL), while Iba-1 immunolabeling was weakly represented in the inner retinal layers. In retinas of PEG-untreated or vehicle-treated mice, Müller cells showed extensive GFAP immunolabeling along their processes spreading across retinal layers and Iba-1 immunoreactivity was more prominent with enlarged amoeboid-shaped immune-positive cells localized in all retinal layers. Treatment with either M1 or M2 at low dose partially prevented Müller cell gliosis and microglial activation as demonstrated by decreased GFAP-positive processes and Iba-1 immunolabeled cells. In retinas of mice treated with M1 at high dose, GFAP and Iba-1 immunoreactivity was less evident, but still detectable. On the contrary, in retinas of mice treated with M2 at high dose, GFAP and Iba-1 immunostaining was not different from control immunostaining. Quantitative analysis of fluorescence intensity showed that in PEG-untreated or vehicle-treated groups, GFAP and Iba-1 immunoreactivity was increased (+440% and +256%, *p* < 0.0001, respectively) as compared to control values. Low dose of M1 or M2 reduced GFAP (-22%, *p* < 0.01) and Iba-1 intensity (−30%, *p* < 0.001) with no difference between the two compounds. Treatment with high dose resulted in a further decrease of GFAP and Iba-1 immunofluorescence, with the high dose of M2 resulting more effective than M1 and preserving GFAP and Iba-1 immunoreactivity to control levels.

### 3.3 Comparative efficacy of the dietary supplementations: Analysis of the proangiogenic marker vascular endothelial growth factor

**FIGURE 5:**
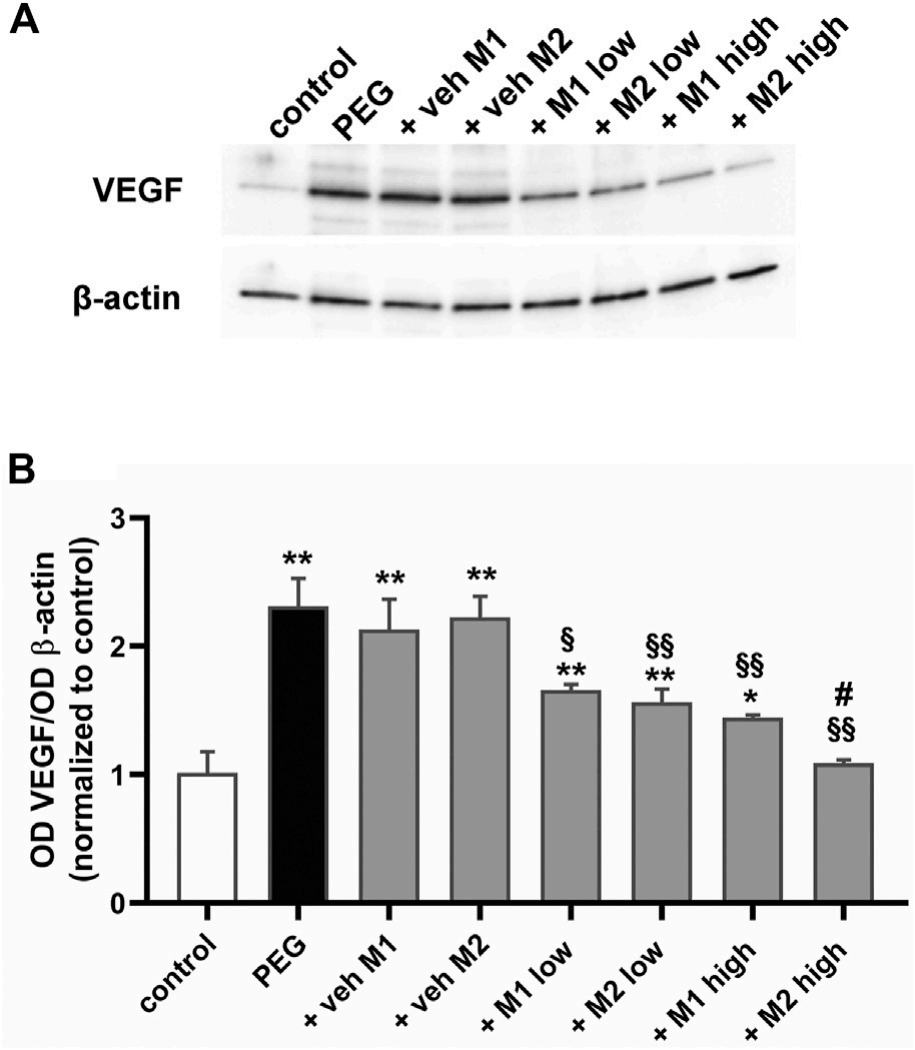
Effect of M1 and M2 on VEGF levels. Representative Western blots **(A)** and densitometric analysis of VEGF **(B)** in controls, PEG-injected untreated, PEG-injected treated with M1 or M2 vehicles, PEG-injected treated with low or high dose of M1 as well as with low or high-dose of M2. β-actin was used as loading control. Data are plotted as mean ± SEM. Differences among groups were assessed using one-way ANOVA followed by Tukey’s multiple comparison post-hoc test (N = 6). *p < 0.01 and **p < 0.001 vs. control; ^§^ *p* < 0.01, ^§§^ *p* < 0.001 vs. respective vehicle; #*p* < 0.05 vs. M1 high.

Pro-inflammatory cytokines are known to increase VEGF expression, which in turn induces a number of pro-inflammatory genes (Schweighofer et al., 2009), indicating that inflammation can promote angiogenesis and new vessel growth thus enhancing tissue inflammation. As shown in Figure 5, PEG-400 injection was found to increase VEGF levels by 131% in respect to the control (*p* < 0.001). Vehicles did not affect VEGF levels while M1 and M2 dose-dependently prevented the PEG-induced VEGF increase with a 26% reduction by both M1 and M2 at low dose. At high dose, M2 was more effective than M1 (−25%, *p* < 0.05 vs. M1) until reaching control levels.

### 3.4 Comparative efficacy of the dietary supplementations: Morphometric analysis of the outer retina

**FIGURE 6:**
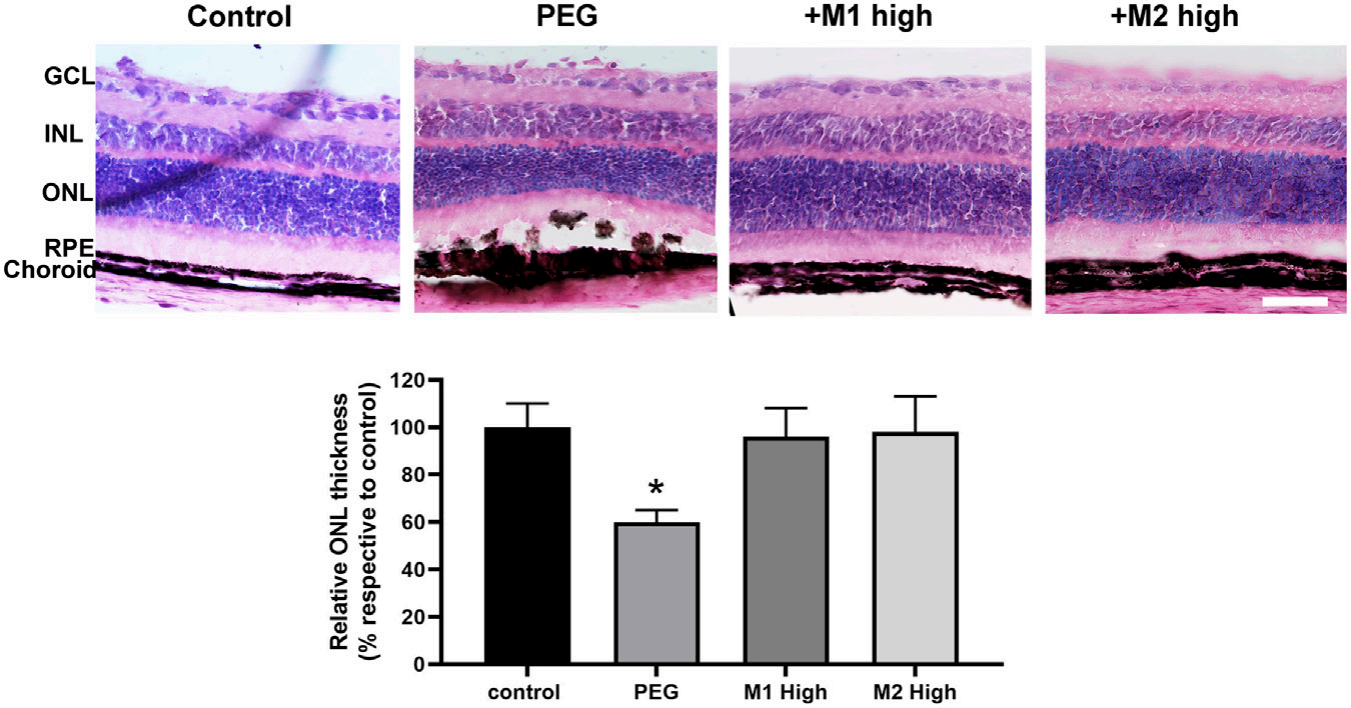
Effect of M1 and M2 on PEG-induced retinal damage. Representative images of H/E-stained retinal cross sections of controls, PEG-injected mice untreated, PEG-injected mice treated with the high doses of either M1 or M2. Morphometric analysis of the ONL thickness normalized to the total retinal thickness and expressed as percentage of controls. Data are expressed as mean ± SEM. Differences among groups were assessed using one-way ANOVA followed by Tukey's multiple comparison post-hoc test (N = 6). *p < 0.05 vs. control. Scale bar, 50 μm. GCL, ganglion cell layer; INL, inner nuclear layer; ONL, outer nuclear layer; RPE, retinal pigment epithelium.

Representative images of H/E-stained retinas are shown in Figure 6 in which histological analysis of the outer retina revealed an evident thinning of the ONL accompanied by the appearance of severe dystrophic alterations of RPE cells upon PEG-400 damage. Dietary supplementation with high doses of both M1 and M2 recovered the control thickness of the ONL including a noticeable recovery of the RPE layer. Quantitative analysis of the ONL thickness revealed that PEG-400 injection leads to a significant reduction of the ONL thickness of about 40% in respect to the control value (*p* < 0.05). High doses of both M1 and M2 were found to lead to ONL thickness almost comparable to that of controls.

## 4 Discussion

Our goal in this piece of translational research was dual: on the one hand, to compare two different, however similar, formulations beyond a direct comparison of the different components in each mixture since their individual role in the protection of the retina is already clear and widely shown, on the other hand, to demonstrate that experimental AMD could be prevented and attenuated by food supplements at doses normally given to humans, and that a significant improvement on the existing formulation is possible by using similar kinds of ingredients, however from different origins. Having in mind the potential antioxidant/anti-inflammatory properties of multicomponent mixtures including xanthophylls and fatty acids, we decided to run a study directly assessing the efficacy of one formulation using fish oil enriched in omega-3 plus lutein and zeaxanthin versus a novel formulation theoretically with a higher efficacy because of a different source of omega-3 and a higher content in xanthophylls. In particular, the novel formulation includes the COil, as an alternative source of omega-3, plus astaxanthin, in addition to lutein and zeaxanthin, whose antioxidant properties in ocular pathologies have been previously demonstrated (Giannaccare et al., 2020; Lombardo et al., 2022). As pharmacological therapies against dry AMD are still lacking, treatments based on potentially innovative dietary supplementations remain as possible tools to solve unmet clinical needs. In an animal model of dry AMD, we established the relative efficacy of both compounds against the PEG-400-induced insult and we found that the COil-based formulation, despite a lower content of omega-3 in respect to that of fish oil origin, had a better protective effect, likely because of the presence of policosanols and the higher amount of additional xanthophylls, now also including astaxanthin beside lutein and zeaxanthin.

In respect to the first Age-Related Eye Disease Study (AREDS) trial demonstrating the efficacy of dietary supplements with antioxidant compounds in reducing the progression to advanced AMD in patients with intermediate AMD (Age-Related Eye Disease Study Research Group, 2001), in the AREDS 2 trial, the addition of lutein and zeaxanthin to the AREDS formula was shown to further decrease the long-term risk of AMD progression (Chew et al., 2022). Conversely, the beneficial effect of the omega-3 fatty acids remained controversial since their addition did not appear to significantly improve the efficacy of the AREDS2 formula (Age-Related Eye Disease Study 2 Research Group, 2013) despite a large background of preclinical and epidemiological evidence favoring the implication of omega-3 in the prevention of AMD progression (Chong et al., 2008). The negative results of the AREDS2 study were then debated in light of the possibility that the prophylactic potential of omega-3 was not adequately demonstrated (Souied et al., 2015). For instance, the need of an “ideal” ratio of omega-3 to omega-6 fatty acids to be assumed in the diet in respect to omega-3 alone, was also recognized in terms of prophylactic benefits as an adequate intake of omega 6 was reported to lower the risk of AMD (Seddon et al., 2003). In this respect, preclinical evidence from the PEG-400 model demonstrated that in addition to omega-3, a correct balance of omega-6/omega-3 is mostly important for preventing dry AMD occurrence and that the efficacy of fatty acids in reducing retinal degeneration may be enhanced by their combination with xanthophylls (Cammalleri et al., 2017). In addition, increasing the amount of fatty acids in the diet would achieve the desired increase in their levels in the body’s tissues with increased effectiveness.

Although the role of omega-3 in AMD still remains controversial, COil, a fatty acid source of copepod origin, raised some interest as a novel source of omega-3 that not only responds to the increasing demand of fatty acids by the market, but also differs from other commercial marine oils in terms of chemical properties and, possibly, bioactivity. In fact, COil contains wax esters, whereas classic omega-3 fatty acids come from triglycerides, ethyl esters and phospholipids. Wax esters are fatty acids that are esterified with alcohols including the long-chain fatty alcohols eicosanol and docosanol that are contained in COil (Schots et al., 2020).

Since early studies in which COil efficacy has been demonstrated in models of human diseases, results of beneficial effects of COil supplementation are rapidly increasing. In this respect, long term intake of COil has been shown to increase the blood level of omega-3 fatty acids despite the low levels of EPA and DHA indicating that fatty acids are mostly of wax ester origin (Wasserfurth et al., 2021). In addition, preclinical findings have indicated antioxidant and anti-inflammatory efficacy of this copepod oil beyond that provided by EPA and DHA (Schots et al., 2020), which are presumably due to fatty acid conversion from their corresponding fatty alcohols (Gasmi et al., 2020). In this respect, notable is the discovery of major effectiveness of COil on glucose metabolism and insulin resistance in obese patients in which the different lipid components of COil may have potential as nutraceuticals for reducing obesity and obesity-related metabolic disorders (Burhop et al., 2022). In mouse models of obesity, COil supplementation in addition to efficiently counteract obesity-induced alterations, has been also demonstrated to exert anti-hypertensive effects and to improve post-ischemic cardiac recovery (Salma et al., 2016; Jansen et al., 2019). In addition, COil supplementation in combination with exercise training has been demonstrated to display additional efficacy on cardiorespiratory function in respect to exercise training alone although its clinical relevance remains to be verified (Štěpán et al., 2021).

The present study comparing the efficacy of two mixtures that differ in their fatty acid source and in their xanthophyll content was performed in the polyethylene glycol- (PEG)-400 model. Although no ideal animal model has been established that fully recapitulates the human features of dry AMD, both the PEG-400 model (Lygozubov et al., 2014) and the chemokine ligand 2 (CCL2) knock out (KO) model (Ambati et al., 2003) share certain common characteristics with dry AMD. Both models contain drusen-like bodies and other features of AMD including progressive outer retinal degeneration and geographic/RPE atrophy although in the CCL2 KO, drusen accumulation consequent to the CCL2 deletion has been also interpreted as a result of normal aging (Luhmann et al., 2009). In both models, dietary supplementation with fatty acids-based mixtures has been found to counteract pro-inflammatory and angiogenic responses, thus hampering the retinal degeneration associated with advanced AMD (Cammalleri et al., 2017; Prokopiou et al., 2017).

The PEG-400 model shows drusen-like deposits that are located between the RPE and the Bruch’s membrane triggering oxidative stress, inflammation and angiogenesis, as key pathways that co-dependently participate in AMD progression. In particular, high levels of reactive oxygen species (ROS) have been associated with drusen formation leading to RPE atrophy in both human and animal eyes (Nowak, 2013; Rabin et al., 2013; Shaw et al., 2016). In this respect, a recent study using a mouse model of AMD has demonstrated an early increase in the expression of oxidative stress and inflammatory markers into the subretinal space and the neural retina (Kim et al., 2021). Chronic inflammation further promotes ROS generation, thus initiating a vicious cycle that promotes the amplification of the pathological events (Datta et al., 2017). The accumulation of ROS triggers the endogenous antioxidant response inducing the activity of nuclear factor erythroid 2-related factor 2 (Nrf2) as the primary redox sensitive transcriptional factor. Nrf2, in turn, mediates the increment in antioxidant enzymes including NQO-1 and HO-1 that are involved in the adaptive response to the altered redox balance (Kang and Yang, 2020). However, the chronic accumulation of ROS overwhelms the antioxidant potential of the endogenous defenses, thus leading to oxidative damage of retinal cells (Kowluru and Chan, 2007). In addition, ROS increase further stimulates the inflammatory response by triggering the activation of NF-kB, a transcription factor primarily involved in the proinflammatory response mediating the expression of cytokines such as IL-6 (Liu et al., 2017). Pro-inflammatory stimuli result in the activation of macroglia, whose switch to reactive phenotype is classically manifested by the overexpression of GFAP. In effect, GFAP expression is widely used as a reliable marker of retinal pathological injury since its localization in the healthy retina is limited to astrocytes, whereas in the diseased retina, GFAP appears densely localized also to Müller cells (Vecino et al., 2016). In this respect, effects of PEG-400 administration are further confirmed by GFAP staining that demonstrates an increase in Müller cell reactivity, as also confirmed by the molecular analysis highlighting the increment in GFAP protein levels. In addition to Müller cell gliosis, over-activated microglial cells participate to the release of pro-inflammatory mediators and further increase oxidative damage (Block and Hong, 2005). Gliotic Müller cells are recognized as the main secretion source of inflammatory cytokines (Eastlake et al., 2016) among which VEGF induces angiogenesis and vascular hyper-permeability thus playing as a major trigger of retinal and choroidal neovascularization leading to wet AMD (Tan et al., 2022). Among the first indications of VEGF role in patients with neovascular AMD, Kliffen et al. (1997) reported its increased expression in human specimens of the RPE and the outer retina at the macular level. The substantial role of VEGF in AMD is sustained by the fact that new therapies for wet AMD are mainly focused on VEGF inhibition although anti-VEGF therapies display significant limitations due to their limited half-life and subsequent treatment costs (Chakravarthy et al., 2022; Sarkar et al., 2022), Therefore, alternative strategies aiming at preventing or delaying the disease progression represents one of the current goals for the management of AMD. In this respect, preclinical studies have demonstrated that treatments with functional nutrients able to inhibit oxidative stress and inflammation may represent a promising approach for non-invasive prevention and/or treatment of AMD. For instance, dietary supplementation based on fatty acids intake has been shown to counteract the production of pro-inflammatory and angiogenic markers thus preventing degenerative events in the outer retinal layers (Cammalleri et al., 2017; Prokopiou et al., 2017). This is confirmed by the present results demonstrating that both mixtures supplemented here exert a multi-target role by interfering with major pathological hallmarks after the PEG-400 insult. In particular, dietary supplementation with fish oil- or COil-based mixtures leads to a consequent reduction of oxidative stress and inflammatory markers thus preventing Müller cell gliosis as determined by the drastically reduced GFAP staining. Microglial reactivity is also prevented as demonstrated by reduced Iba-1 at the immunohistochemical and protein level. Preventing oxidative stress and inflammatory processes can be reflected in counteracting VEGF upregulation typical of AMD-like conditions, thus suggesting a possible inhibition of the switch from dry to wet AMD. At the structural level, dietary supplementation with both mixtures promotes the recovery of the ONL thickness, with clear benefits to the RPE cells.

Most importantly, the present findings confirm the efficacy of an oral delivery of lutein and omega-3 fatty acids in the prevention of dry AMD, and likely its progression towards the wet form. Such efficacy can be further improved by a novel formulation based on a different source of omega-3 fatty acids (the COil) also containing policosanols, and an increased amount of xanthophylls, namely astaxanthin in addition to lutein. A contribution to this higher efficacy might be ascribed to the increased bioavailability of omega-3 in the COil formulation (Cook et al., 2016) and its content in astaxanthin with higher antioxidant activity as compared to lutein and zeaxanthin (Naguib, 2000). Increased availability of omega-3 fatty acids might depend on their partial origin from policosanols, which could restore with higher efficacy the deficiency of bioactive lipids as those derived from polyunsaturated fatty acids that are known to play a protective role against AMD development (Kishan et al., 2011; Elmasry et al., 2019). Moreover, despite COil is relatively low in EPA and DHA as compared to fish oil, its high content in the monounsaturated SDA, a precursor of EPA and DHA, may further contributes to increase omega-3 fatty acid content (Pedersen et al., 2014; Cook et al., 2016).

## 5 Conclusion

Nowadays, there is no cure for AMD, much less for its atrophic form. The neovascular form can be treated by intravitreal anti-inflammatory and/or anti-angiogenic drugs. Antioxidant xanthophylls and carotenoids are used to delay progression of the atrophic form, which may degenerate into the neovascular disease. Experimental therapies, mainly based on the control of oxidative stress, are under study including clinical trials using compounds that reduce mitochondria-derived oxidants.

Among dietary supplementations eventually counteracting dry AMD, the AREDS studies have clearly indicated the role of diets containing antioxidants and omega-3 in the prevention and control of the progression of AMD. Among structural components of retinal cell membranes, cholesterol mostly of Muller glia origin is the second most abundant lipid in the neuroretina behind phospholipids. Cholesterol plays a key role in retinal function and its downregulation can contribute to the progression of several multifactorial diseases like glaucoma, diabetic retinopathy and macular edema. A lipid shuttle has been identified from Müller glia to retinal ganglion cells to sustain their projections and synaptic formation. Whether fatty acids supplementation would cover the increasing demand of fatty acids by retinal neurons, then nutritional supplementation with omega-3 may represent an important coadjuvant in the therapeutic management of AMD.

Among antioxidants, carotenoids accumulate in the macula that naturally contains antioxidants and photo-bleachers such as lutein and zeaxanthin. Astaxanthin, which is even more potent than lutein, is not naturally present at the macular level. Therefore, the main, if not the only, treatment for the prevention and the treatment of the early forms of AMD is the supply of nutraceuticals to replenish the antioxidant defense of the retina and to give anabolic support to photoreceptors. In this respect, individuals with high risk of developing AMD, as predictable with an available genetic test, might be advised to use more antioxidants and omega-3 in their diet.

Results presented in this paper show the relevance of the combination of dietary omega-3 and xanthophylls in the prevention and treatment of AMD and indicate that the source of these components might be also critical, because the formulation based on COil appears to be more protective than the one based on fish oil. In this respect, innovative dietary treatments including fatty acids of COil origin and xanthophylls may potentially represent AMD modifying therapies with higher efficacy than currently used therapies including fatty acids of fish oil origin.

## Data Availability

The raw data supporting the conclusions of this article will be made available by the authors, without undue reservation.
