# Correction: Increased efficacy of dietary supplement containing wax ester-rich marine oil and xanthophylls in a mouse model of dry macular degeneration

**DOI:** 10.3389/fphar.2026.1850473

**Published:** 2026-05-08

**Authors:** Alberto Melecchi, Rosario Amato, Dominga Lapi, Massimo Dal Monte, Dario Rusciano, Paola Bagnoli, Maurizio Cammalleri

**Affiliations:** 1 Department of Biology, University of Pisa, Pisa, Italy; 2 Interdepartmental Research Center Nutrafood “Nutraceuticals and Food for Health”, University of Pisa, Pisa, Italy; 3 Research Center, Fidia Farmaceutici S.p.A., Catania, Italy

**Keywords:** calanus oil, omega-3 fatty acids, carotenoids, oxidative stress, inflammation, gliosis, retinal thickness

There was a mistake in Figure 6 as published. Specifically, the image for the control group was incorrectly selected during figure preparation. The affected image has been replaced with the correct representative image from the original experiments. These changes do not affect the scientific conclusions of the article in any way. The corrected Figure 6 appears below.

**FIGURE 6:**
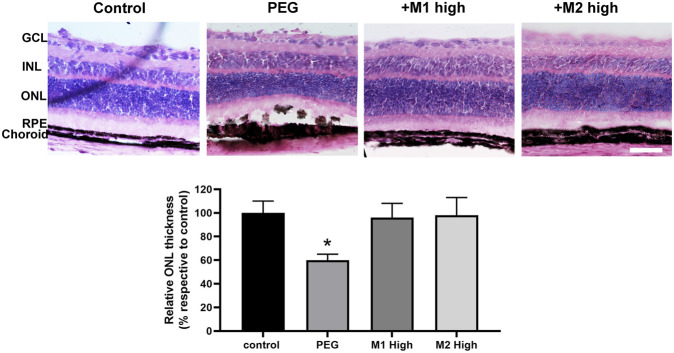
Effect of M1 and M2 on PEG-induced retinal damage. Representative images of H/E-stained retinal cross sections of controls, PEG-injected mice untreated, PEG-injected mice treated with the high doses of either M1 or M2. Morphometric analysis of the ONL thickness normalized to the total retinal thickness and expressed as percentage of controls. Data are expressed as mean ± SEM. Differences among groups were assessed using one-way ANOVA followed by Tukey’s multiple comparison *post hoc* test (N = 6). *p < 0.05 vs. control. Scale bar, 50 μm. GCL, ganglion cell layer; INL, inner nuclear layer; ONL, outer nuclear layer; RPE, retinal pigment epithelium.

The original article has been updated.

